# A Robust Masked Painter Framework for Gene Selection in Binary Classification of High-Dimensional Functional Genomic Data

**DOI:** 10.3390/e28090992

**Published:** 2026-09-04

**Authors:** Sehran Hassan, Alamgir, Hasnain Iftikhar, Asma Gul, Abdur Rehman, Paulo Canas Rodrigues

**Affiliations:** 1 Department of Statistics, University of Peshawar, Peshawar 25120, Pakistan; sehranhassan@uop.edu.pk (S.H.); alamgir_khalil@uop.edu.pk (A.); abdurrehmanshad@uop.edu.pk (A.R.); 2 NUST Business School, National University of Sciences and Technology, Islamabad 44000, Pakistan; asma.gul@nbs.nust.edu.pk; 3 Department of Statistics, Federal University of Bahia, Salvador 40170-110, Brazil; paulo.canas@ufba.br; 4 Department of Business Management, University of Pretoria, Pretoria 0002, South Africa

**Keywords:** gene selection, high-dimensional data, robust feature selection, machine learning, robust statistics, biomedical data analysis

## Abstract

High-dimensional gene expression datasets in chemometric and biomedical research present significant challenges for machine learning because the number of genes greatly exceeds the number of available samples, increasing the risk of overfitting and reducing classification reliability. Existing gene selection methods are often sensitive to noise and outliers, leading to unstable feature subsets and degraded classification performance. To address these limitations, this study proposes a Robust Masked Painter (RMP) framework that integrates robust measures of location and dispersion, namely the Median and the Rousseeuw & Croux statistic ($Q_{n}$), for reliable gene selection. The proposed framework operates in two stages. First, we identify informative genes using a round-robin strategy with a greedy search algorithm and robust core intervals to reduce the influence of noise and outliers. Second, Dominant Class (DC) analysis and Overlapping Scores (*OS*) further refine the selected gene subset by minimizing class overlap. We evaluate the proposed method on four publicly available gene expression datasets and compare it with several established feature selection methods using Random Forest, K-Nearest Neighbors, and Support Vector Machine classifiers. We assess classification performance using the Classification Error Rate. Experimental results and simulation studies demonstrate that the proposed RMP framework consistently outperforms competing methods by selecting highly informative genes that improve classification accuracy, robustness, and generalization.

## 1. Introduction

Microarray and other high-throughput functional genomics technologies have become important tools for investigating gene-expression patterns and identifying molecular characteristics associated with different biological conditions [1,2,3]. However, gene-expression datasets are typically high-dimensional, with the number of genes substantially larger than the number of available samples. Many genes may be irrelevant, redundant, or only weakly associated with the response, while measurement noise and outlying observations can further obscure the underlying discriminative patterns [4,5,6,7]. Consequently, identifying a compact, reliable subset of informative genes is an important step toward improving classification performance, reducing computational complexity, and facilitating biological interpretation of predictive models [8,9,10].

Machine learning (ML) methods, including Support Vector Machines (SVMs), Random Forests (RFs), and K-Nearest Neighbors (K-NN), have been widely used to classify high-dimensional gene-expression data [11]. However, their performance can be strongly affected by input dimensionality and feature quality. Feature selection offers an effective strategy to address these challenges by retaining informative genes while removing irrelevant and redundant variables. Unlike dimensionality-reduction approaches, feature selection preserves the original biological meaning of the selected genes. Existing feature-selection approaches generally fall into filter, wrapper, embedded, or hybrid methods [12,13,14,15]. Filter methods commonly rank genes using statistical criteria such as the *t*-test, Wilcoxon test, or Mann–Whitney test [16,17,18,19,20]. Although computationally efficient, these methods often evaluate genes individually and may not adequately account for complex relationships among features. Wrapper methods evaluate candidate subsets through predictive models and can therefore identify subsets based on prediction performance, but their computational cost becomes substantial in high-dimensional settings. Embedded approaches, such as tree-based methods and stepwise regression, perform feature selection during model estimation and can be more computationally efficient; however, they may still be affected by overfitting, multicollinearity, or model-specific assumptions [21,22,23,24,25,26,27,28,29,30,31,32,33].

Another challenge in gene-expression analysis is noise and outliers. Classical statistical measures used for gene ranking can be substantially influenced by extreme observations, potentially leading to unstable rankings and the selection of non-informative genes. Several approaches have therefore incorporated robust statistics into feature selection. Researchers have investigated correlation- and consistency-based approaches to identify stable feature subsets [34,35], and have also proposed optimization-based approaches, including ant colony optimization and clustering-based strategies [36,37,38]. More recently, Proportional Overlapping Score (POS), Robust Proportional Overlapping Score (RPOS), and related robust feature-selection procedures have been developed to improve gene discrimination under noisy conditions [34,38,39,40,41]. These methods provide improved resistance to contamination; however, their performance can still deteriorate when the proportion of outliers increases or when the distributions of the two classes exhibit substantial overlap. Recent gene-selection studies have also explored weighted statistical scores, support-vector-based sample selection, and sequential feature-selection strategies to obtain compact and discriminative subsets [42,43,44]. These developments demonstrate the importance of robust and discriminative feature-selection mechanisms for high-dimensional biomedical data, but also highlight the need for methods that can simultaneously control the influence of outliers and distinguish informative genes under overlapping class distributions.

Motivated by these limitations, this study proposes a Robust Masked Painter (RMP) framework for gene selection in binary classification of functional genomics data. The proposed framework incorporates the median and the Rousseeuw–Croux $Q_{n}$ estimator as robust measures of location and dispersion, respectively, thereby reducing the influence of extreme observations during gene selection. The method operates through a two-stage selection procedure. First, a round-robin strategy combined with a greedy search and robust core intervals identifies potentially informative genes while limiting the influence of noisy observations. Second, Dominant Class (DC) analysis and Overlapping Scores (OS) refine the candidate subset based on its ability to discriminate between the two classes. This design aims to obtain compact, robust, and stable gene subsets, particularly when the data contain contamination or overlapping class distributions.

This study makes three main contributions. First, we develop a robust gene-selection framework by integrating the median and Rousseeuw–Croux $Q_{n}$ estimator into the selection process. Second, we introduce a two-stage selection mechanism that combines robust core intervals, round-robin greedy search, Dominant Class analysis, and Overlapping Scores to identify informative and discriminative genes. Third, we evaluate the proposed method using four publicly available gene-expression datasets and controlled simulation scenarios with different contamination levels. We compare its performance with established feature-selection methods using Random Forest, K-Nearest Neighbors, and Support Vector Machine classifiers, using Classification Error Rate as the primary evaluation criterion.

The rest of the paper is organized as follows. Section 2 describes the proposed Robust Masked Painter framework. Section 3 presents the benchmark gene-expression datasets, experimental settings, and evaluation metrics. Section 4 presents the simulation study, experimental results, statistical analysis, and comparison with existing feature-selection methods. Finally, Section 5 concludes the paper and discusses directions for future research.

## 2. Proposed Methodology

This section defines the methods and datasets used in the present study. The classifiers categorize data points into established classes, such as the presence or absence of the disease, which is a binary classification problem. Microarray technology can capture thousands of genes; however, it often produces noisy, redundant data, making feature selection crucial for identifying informative genes that improve classification accuracy [23,45]. Given the high-dimensional characteristics of microarray datasets, sophisticated computational procedures are essential for effective analysis. Feature selection is vital for improving both computational efficiency and prediction accuracy [46].

### 2.1. Masked Painter

The Masked Painter (MP) technique, inspired by Painter’s algorithm in computer graphics, prioritizes genes based on overlaps in their expression intervals. It has two key advantages: (i) it identifies the smallest subset of genes for optimal classification, covering the data efficiently, and (ii) it assigns quality scores to genes via a density-based approach to reduce the impact of outliers. The final feature set integrates the smallest subset of genes with top-ranking genes based on their quality scores. The word Masked denotes the organized gene information format that facilitates overlap analysis and prioritization [47].

### 2.2. Proposed Robust Masked Painter

The proposed Robust Masked Painter (RMP) framework is developed as a general feature selection strategy for high-dimensional binary classification problems. Although the present study focuses on functional genomics data, the framework is not restricted to gene expression data. It applies to any binary classification problem in which a large number of predictors are measured for a relatively small number of observations. The central principle of RMP is to represent each predictor through its class-specific expression intervals, quantify its ability to uniquely cover training observations, and subsequently rank predictors according to robust class separation and overlap. Let the predictor matrix be represented by

(1) $$U=\left[\begin{array}{cccc} e_{11} & e_{12} & \cdots & e_{1M}\\ e_{21} & e_{22} & \cdots & e_{2M}\\ \vdots & \vdots & \ddots & \vdots \\ e_{N1} & e_{N2} & \cdots & e_{NM} \end{array}\right]$$

As shown in the above matrix (1), let $e_{ij}$ represent the expression level of gene *i* in sample *j*, where i=1,2,…,M and j=1,2,…,N denote the gene and sample indices, respectively. Each sample is assigned a class label that reflects the clinical condition of the patient or tissue under investigation. The set of class labels consists of distinct values, and each sample is assigned one specific label from this set.

**Figure 1:**
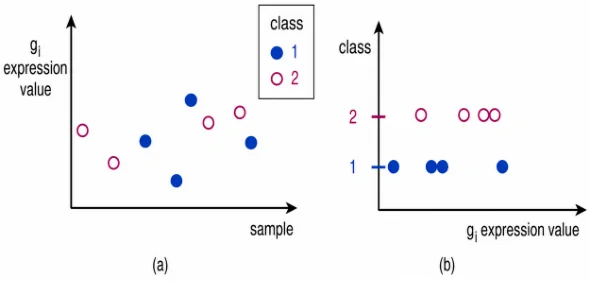
The expression profile of a gene using two representations such as, (**a**) gene vs. sample; (**b**) gene vs. class.

Figure 1 depicts a gene expression profile using two distinct representations across eight samples, each associated with one of two classification categories.

**Figure 2:**
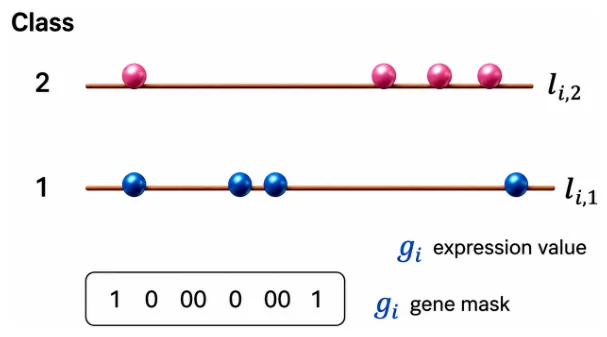
Gene’s mask and expression intervals.

**Figure 3:**
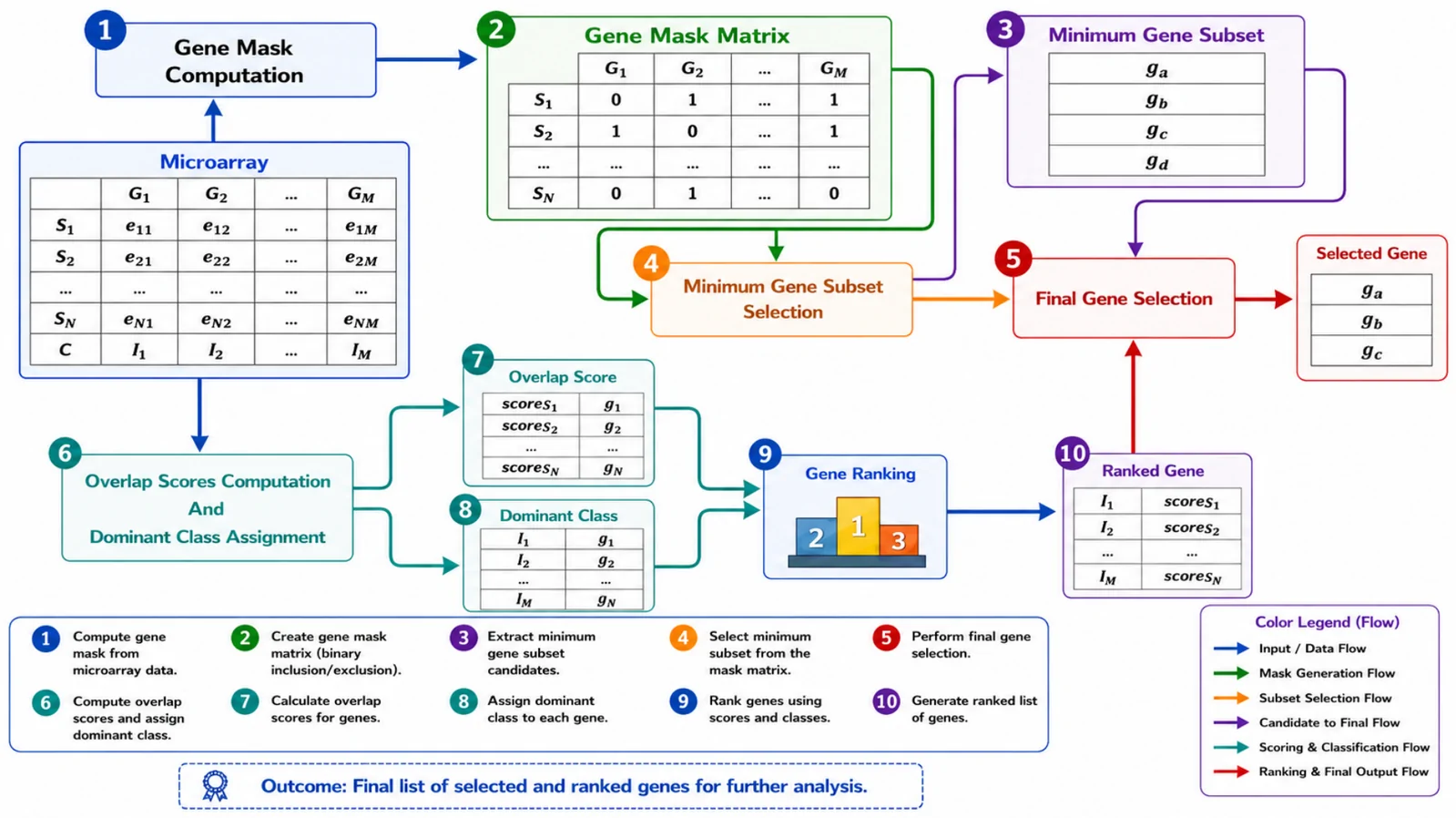
Workflow of the Masked Painter method [47].

Figure 2 illustrates a gene’s mask and its class-specific expression intervals across eight samples distributed between two distinct classes, and Figure 3 shows the detailed workflow of the Masked Painter method.

### 2.3. General Principle and Reproducibility of the RMP Framework

The proposed Robust Masked Painter (RMP) framework is not an ad hoc empirical procedure; rather, it is a general and reproducible feature selection rule defined by a sequence of mathematically specified operations. The underlying principle is to select genes according to three complementary criteria: (i) the ability of a gene to uniquely identify training samples through its gene mask, (ii) the degree of overlap between the robust class-specific expression intervals, and  (iii) the balance of discriminative information across classes.

For a gene *i*, its discriminative information is first represented by the binary mask $Z_{i}$, which identifies the training samples that can be uniquely associated with a class. The minimum gene subset is then constructed by maximizing the incremental coverage of the training samples. Specifically, at each iteration, the gene that provides the largest number of previously uncovered samples is selected. When two or more genes provide identical coverage, the gene having the smallest overlap score is selected. Thus, the selection criterion can be expressed as the following lexicographic rule:

(2) $$i^{*}=\arg\max_{i\in F}\left(\sum_{j=1}^{N}Z_{ij},-{\mathrm{OS}}_{i}\right),$$

where F denotes the set of candidate genes and ${\mathrm{OS}}_{i}$ represents the overlap score of gene *i*.

The robustness of the framework is introduced through the use of the sample median and the Rousseeuw–Croux $Q_{n}$ estimator when constructing the core expression intervals. Consequently, the estimation of the location and dispersion of gene expression is not determined by potentially influential extreme observations. The resulting core intervals provide a consistent basis for calculating the overlap score and dominant class for every gene.

The complete RMP procedure is therefore determined by explicit mathematical rules: gene masks define sample coverage, the greedy criterion determines the minimum discriminative subset, the robust core intervals determine resistance to contamination, the overlap score quantifies class overlap, and the dominant-class rule ensures representation of both classes. Given the same training data, class labels, and predefined selection parameters, these deterministic rules produce the same selected gene subset. This formulation makes the proposed framework reproducible and allows the RMP principle to be applied to other high-dimensional binary classification problems beyond the gene-expression datasets considered in this study.

### 2.4. Gene Mask Calculation

To capture each gene’s discriminative power, we introduced a concise representation called the gene mask. A gene’s discriminative power indicates its ability to accurately categorize each sample. The class expression intervals for every gene are established by considering the gene expression matrix *U*, which includes a class label for each sample. Let us suppose that *i* is an arbitrary gene and that *N* samples come from *k* classes. We define *k* class expression intervals (one for each class) for every gene *i*. Given the expression intervals for gene *i* and class *k*, classes are represented as follows:

(3) $$I_{i,k}=\;\left[{Min}_{i,k},{\;\;\;\;Max}_{i,k}\right]$$

For each class *k*, ${Min}_{i,k}\;\mathrm{and}{\;Max}_{i,k}$ represent the minimum and maximum gene expression levels, respectively. To preserve the natural range of the training data, the gene mask exploitation approach relies directly on this min–max interval. We apply no additional preprocessing, such as outlier adjustment or smoothing. A gene mask is a binary array of length *N*, where *N* is the number of samples. Consider a random gene *i* for each sample *j*. The expression value $e_{ij}$ is compared against the class-specific expression intervals. If $e_{ij}$ falls within the interval of exactly one class, thus unambiguously associating the sample with that class, the $j^{th}\;$ bit of the mask for gene *i* is assigned a value of 1. Otherwise, set the bit to 0. Formally, for a two-class problem, the $j^{th}$ bit of the mask for gene *i* can be computed as follows, where $a,\;b\;\in \;\left\{1,\ldots ,k\right\}.$

(4) $${Mask}_{ij}\;=\;\left\{\begin{array}{c} 1\;\;if\left(e_{ij}\;\in \;I_{i,a}\right)\;\wedge \;\exists b\;\neq \;a/e_{ij\;}\;\in \;I_{i,b}\;\\ \;\;\;\;\;\;0\;\;\;\;\;\;\;\;\;\;\;\;\;\;\;\;\;\;\;\;\;\;\;\;\;\;Otherwise \end{array}\right.\;$$

This is done following the derivation of the class-specific expression intervals $(I_{i,1}$, $I_{i,2}).$ The gene mask associated with gene *i* is depicted in Figure 2. The gene mask therefore provides a general representation of each gene’s sample coverage. A value of one indicates that the corresponding expression observation is uniquely identifiable by the class-specific intervals, whereas a value of zero indicates ambiguity or insufficient discriminative information. Consequently, the logical union of gene masks provides a natural measure of the cumulative classification coverage achieved by a selected gene subset. During selection of the minimal gene subset, gene masks identify the features that provide the most comprehensive coverage of the training samples.

### 2.5. Overlap Score Computation and Dominant Class Assignment

An *OS* is assigned to every gene, determined by the extent of overlapping expression intervals between classes. Unlike the gene mask, which is based on min–max expression intervals, the *OS* models genes’ discriminative capability and must handle noise and outliers to prevent overfitting. As a result, to enhance the modeling of the expected values in an unseen set, the *OS* is computed based on core expression intervals. Core expression intervals are those obtained by mitigating the impact of outliers through a density-based approach. The class with the most samples in non-overlapping intervals, called the dominant class, is likewise assigned to each gene. This phase is further categorized into three steps, which are explained below: (i) core expression interval definition, (ii) overlap score computation, and (iii) dominant class assignment.

#### 2.5.1. Core Expression Interval Definition

Microarray datasets often include noise, which methods such as the 3-sigma rule and the Hampel identifier can reduce [20,47]. A robust approach reduces the effect of outliers when determining a gene’s core expression interval. Instead of depending on the mean and standard deviation, this method substitutes them with the median and the Rousseeuw and Croux statistic. These robust statistics provide a more reliable estimate of the gene’s central tendency and variability, ensuring outliers do not distort the interval. This approach simulates the possible distribution of values for unseen (test) expressions. For a certain expression value, the density weight $d_{ij}$ measures the quantity of nearby expression values derived from samples that belong to the same class. Consider an arbitrary sample *j* from class *k* and the expression value of its arbitrary gene *i*, denoted as $e_{ij}$. Assume that the expression values are modeled as independent and identically distributed (i.i.d.) random variables, and let $Q_{n_{\left(i,k\right)}}$ denote the Rousseeuw and Croux statistic for gene *i* expression values in class *k*. For the specified expression value $e_{ij},\;$ the density weight $d_{ij}$ assesses how many expression values from samples of the same class are located within its vicinity. In class *k*, the density weight for $e_{ij}$ is defined as:

(5) $$d_{ij}\;=\;\sum_{n=1,\;n\neq j}^{N}Q_{(n)in}\;\;$$

where $Q_{(n)in}$ is defined as

(6) $$Q_{(n)in}=\;\;\left\{\begin{array}{c} 1\;\;if\;sample\;n\;belongs\;to\;class\;k\wedge \;e_{in}\in \;\left[e_{ij}\;-\;Q_{(n)i,k}\;\;\;\;\;\;;\;e_{ij}\;+\;Q_{(n)i,k\;}\right]\\ 0\;\;\;\;\;\;\;\;\;\;\;\;\;\;\;\;\;\;\;\;\;\;\;\;\;\;Otherwise \end{array}\right.$$

**Figure 4:**
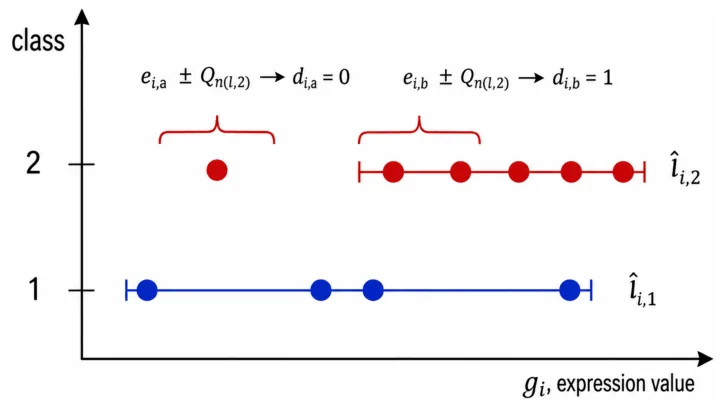
The core expression interval of a gene utilizing density weights.

An expression value attains a higher density when it is accompanied by nearby values from the same class. As illustrated in Figure 4, gene *i* consists of 10 samples distributed across two classes. The first class 2 sample (represented as $e_{ia}$ in Figure 4) is categorized by a density weight $d_{ia}=$ 0, because there are no other expression values for gene *i* in the interval $e_{ia}\pm {Q_{n}}_{i,2}$. The other class 2 sample ($e_{ib}$) is defined instead by a density weight $d_{ib}=$ l, because other samples of class 2 fall within the interval $e_{ib}\pm {Q_{n}}_{i,2}$. The core expression interval for the gene i in class k is defined as:

(7) $${\hat{I}}_{i,k}\;=\;{Med}_{i,k}\;\pm \;2*Q_{n_{\left(i,k\right)}}$$

where the ${Med}_{i,k}$ is the median and $Q_{n_{\left(i,k\right)}}$ is the Rousseeuw and Croux statistic.

The use of $Med_{i,k}$ and $Q_{n(i,k)}$ establishes the robustness principle of RMP. In contrast to mean–standard deviation intervals, the location and scale estimates used here have reduced sensitivity to extreme observations. Therefore, the same interval construction rule is applied uniformly to every gene and class, providing a general robust mechanism for identifying the central expression region.

(8) $$Q_{n(i,k)}\;=\;c_{n(i,k)}*Q_{1(i,k)}\left(\left|e_{l(i,k)}-\;e_{p(i,k)}\right|\right)$$

where $C_{n(i,k)}$ represents a constant that is influenced by the size of the *i^th^* gene and the class *k*. The value $\left|e_{l(i,k)}-\;e_{p(i,k)}\right|$ indicates the absolute pairwise differences in gene expression values for the *i^th^* gene, for classes *k* = 0 or 1. Figure 4 shows the core expression interval of genes utilizing the density weights.

#### 2.5.2. Overlap Score Computation

An *OS* is calculated for every gene to assess the degree of overlap among core expression intervals. Because overlapping intervals can cause misclassification when a gene has insufficient discriminative capacity, the *OS* is used to rank genes. Genes with extensive overlap between class intervals are considered less significant and receive higher scores. Conversely, genes with minimal overlap are better at separating classes and are assigned lower *OS* values. The *OS* depends on the subsequent characteristics of the gene expression values: (i) The number of samples from different classes that exist within the identical range. (ii) The degree of class overlap. (iii) The extent of the overlapping interval. The $OS_{i}$ is computed for each gene. To restructure notation in the formulas, we will eliminate the subscript i.

We outline the expression interval of a gene as the span between the minimum and maximum limits of its total expression intervals. This interval is represented by *W*, with its length denoted by |W|. We split *W* into smaller sub-intervals, each defined by a different set of overlapping classes in relation to its adjacent intervals. Specifically, the subinterval $w_{t}$ is identified as the interval marked by two consecutive extremes of core expression intervals. The amplitude of the subinterval $w_{t}$ is expressed as |$w_{t}$|. Utilizing this mechanism, the *OS* for any gene can be formally articulated.

(9) $${OS}_{i}\;=\;\sum_{t=1}^{T}k(t)\;\;\frac{m_{t}\;\;}{N}\;\;\;\frac{\left|w_{t}\right|}{\left|W\right|}$$

where N stands for the overall number of examples, *t* is the number of sub-intervals, and $m_{t}$ represents the quantity of samples located within sub-interval *t*. The next section describes the mathematical expression for k(t), which counts the number of classes whose values intersect within sub-interval *t*.

(10) $$k\left(t\right)\;=\;\;\;\left\{\begin{array}{c} \left|C_{t}\right|\;\;\;\;\;if\;\left|C_{t}\right|\;\;\geq \;\;2\\ 0\;\;\;\;\;Otherwise \end{array}\right.$$

**Figure 5:**
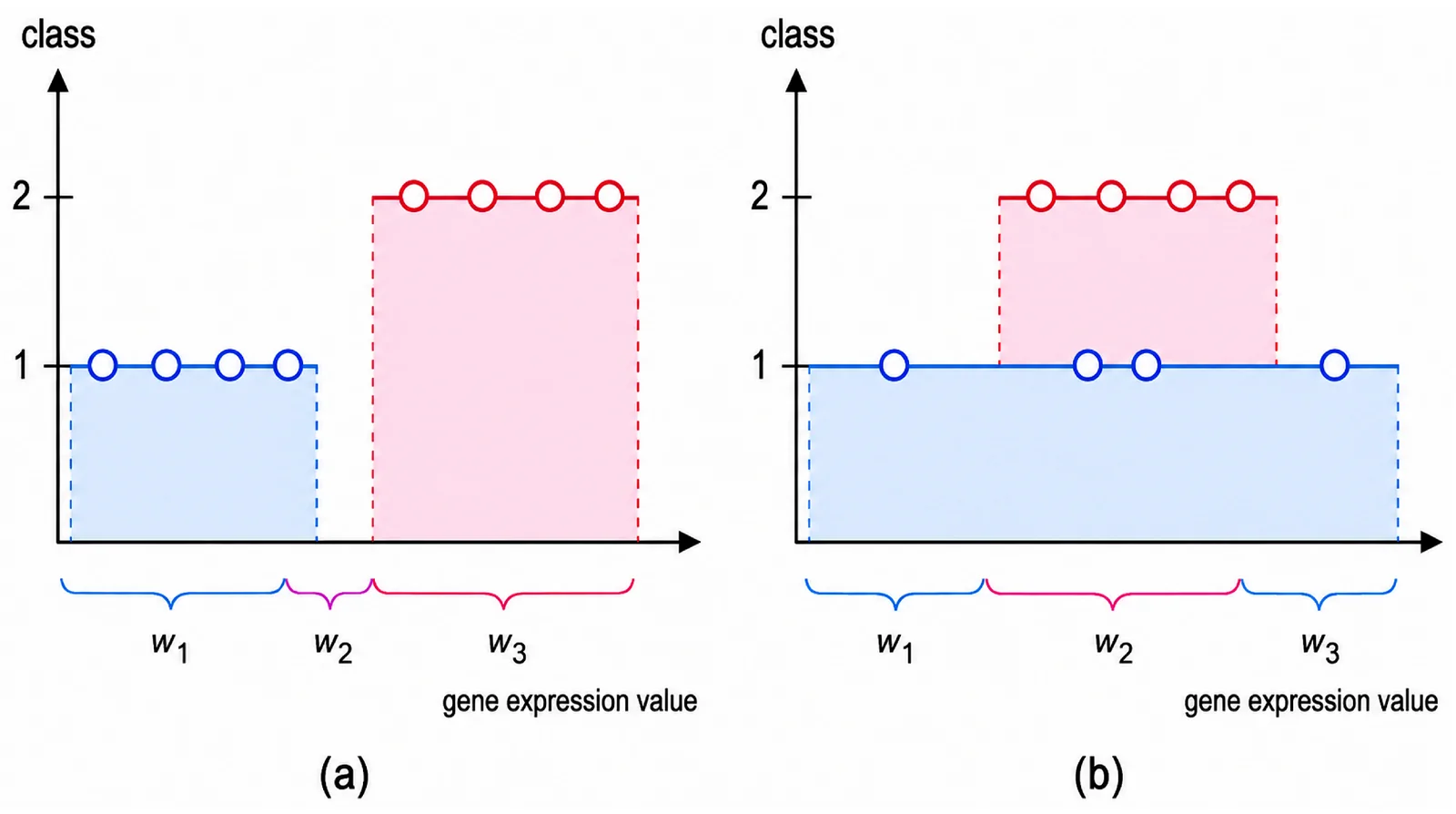
Overlap score calculation: (**a**) Overlap score of 0. (**b**) Overlap score close to 2.

The quantity $\left|C_{t}\right|\;$ is the number of classes with samples in sub-interval *t*. A gene with no overlap (Figure 5a) has *OS* = 0; full overlap (Figure 5b) gives *OS* ≈ 2.

A gene that accurately separates two classes is shown in Figure 5a, as its core expression intervals remain non-overlapping. For this gene, the *OS*-value is equivalent to zero. Rather, Figure 5b depicts a gene that fails to distinguish between two groups as a result of the significant overlap in the expression interval associated with both classes. In this situation, the *OS*-value is relatively close to 2.

#### 2.5.3. Dominant Class Assignment

Once we calculate the *OS*, we assign each gene to the class it distinguishes most effectively, its DC. To do this, we examine subintervals where the expressed samples fall into a single class and assess the percentage of samples belonging to the specified class within those subintervals. The class with the most samples is deemed the DC for the gene. The gene is assigned to the class with the most samples, considering the a priori class probabilities. By connecting a gene to the class that differentiates it most accurately, we can ensure a balanced selection of genes across classes.

### 2.6. Minimum Gene Subset Selection

The gene masks determine the smallest group of genes that accurately classifies the largest number of samples in the training set. It also enables the elimination of redundant information (such as genes with similar expression patterns or low discriminative power). The ultimate goal is to identify a set of high-quality genes that effectively represents the given sample set. Let *S* represent a set of genes. A *global-mask* is identified as the logical OR of all gene masks associated with the genes in *S*. The objective of this research is to determine the minimum subset of genes *S* that possesses sufficient discriminating ability to allocate the maximum number of training examples to their appropriate classes. Therefore, the goal is to identify the global mask with the most ones based on each gene’s mask. The minimum gene subset selection follows a general maximum-coverage principle. Let $E^{\left(r\right)}$ denote the selected gene subset at iteration *r* and let $G^{\left(r\right)}$ denote its corresponding global mask. For a candidate gene *i*, its incremental coverage at iteration *r* is defined as

(11) $$\Delta_{i}^{\left(r\right)}=\sum_{j=1}^{N}Z_{ij}\left(1-G_{j}^{\left(r\right)}\right),$$

where $Z_{ij}$ is the *j*th element of the gene mask for gene *i* and $G_{j}^{\left(r\right)}$ indicates whether sample *j* has already been covered by the selected genes.

The next gene is selected according to

(12) $$i^{\left(r+1\right)}=\arg\max_{i\in F^{\left(r\right)}}\Delta_{i}^{\left(r\right)},$$

where $F^{\left(r\right)}$ is the set of remaining candidate genes. If  multiple genes attain the same maximum incremental coverage, the tie is resolved by selecting the gene with the minimum overlap score:

(13) $$i^{\left(r+1\right)}=\arg\min_{i\in T^{\left(r\right)}}OS_{i},$$

where $T^{\left(r\right)}$ contains the genes attaining the maximum incremental coverage.

Thus, the selection mechanism is fully specified by a deterministic optimization and tie-breaking rule rather than by subjective or empirical choices.

A feasible technique, namely the Greedy Search Method, is employed to achieve this objective. At each iteration, the greedy search algorithm selects the gene that contributes the most effective integrated gene mask relative to the current global mask. Consequently, it provides enough information to classify most currently unclassified training examples at each stage. Algorithm 1 presents the pseudo-code of the Greedy Search Method. The algorithm takes the collection of overlap scores (OS) and the set of gene masks (*Z*) as inputs and returns the minimum subset of genes (E). Initially, the selected gene subset is initialized as E=∅ (Line 2), the candidate gene set as F=∅ (Line 3), and the global mask as an all-zero vector (Line 4). The remaining steps are then performed iteratively as follows:

i.   Line 8 identifies the candidate gene mask containing the largest number of ones. If multiple gene masks satisfy this criterion, select the gene with the minimum overlap score (Lines 9–16).
ii.  The selected gene is added to the subset E (Line 18), and the global mask is updated (Line 19) by performing the logical OR operation with the selected gene mask.
iii. Using the complement of the updated global mask (Lines 21–24), a logical AND operation is applied to revise the remaining gene masks in the set *Z* (Line 20). Consequently, the algorithm retains only those mask positions corresponding to training examples that remain unclassified for subsequent iterations.
iv. The algorithm terminates when either the global mask contains no zero entries (Line 6), indicating complete coverage of the training samples, or no remaining candidate gene contains any uncovered ones (Line 9).

This procedure concludes by identifying the minimum gene subset required to maximize coverage of the training data. The genes in the resulting subset are ordered by the decreasing number of ones in their corresponding gene masks.
**Algorithm 1** Minimum Gene Subset—Greedy Approach**Require:** Set *Z* of all masks $Z_{i}$, overlap score set OS containing $OS_{i}$ for each gene *i***Ensure:** Selected gene subset E  1:E←∅  2:F←∅                  ▹ Candidate gene set at each iteration  3:globalMask←0                   ▹ Vector containing only zeros  4:**while** globalMask is not all ones **do**  5:      F← genes having the maximum number of ones  6:      **if** F≠∅ **then**              ▹ Pick the candidate with the minimum overlap score  7:             f←F[1]  8:             **for all** j∈F[2:] **do**  9:                   **if** $OS_{j}<OS_{f}$ **then**10:                         f←j11:                   **end if**12:             **end for**                     ▹ Update selected genes and global mask13:             E←E∪{f}14:             $\mathrm{globalMask}\leftarrow \mathrm{globalMask}\vee Z_{f}$15:             $Z\leftarrow Z-Z_{f}$                            ▹ Update remaining masks16:             **for all** $Z_{i}\in Z$ **do**17:                   $Z_{i}\leftarrow Z_{i}\wedge \neg \left(\mathrm{globalMask}\right)$18:             **end for**19:      **else**20:             **break**21:      **end if**22:**end while**23:**return** E

### 2.7. Gene Ranking

The ranking considers both *OS* and DC. Genes outside the minimum subset are ranked individually for each DC by increasing overlap score. The final rank is formed by selecting the top gene from each DC rank in a round-robin approach. Ref. [48] introduced a feature-selection method that focuses solely on a simplified version of the *OS*. The *OS* independently ranks high genes with little overlap, neglecting the class it distinguishes best. Thus, feature selection may become biased, as highly ordered genes classify all examples as belonging to a single class (usually by excluding less populous categories). The DC’s round-robin selection process can counter this.

### 2.8. Final Gene Selection

Genes are ordered by a decreasing number of ones in their gene masks; the minimum gene subset is the smallest group of genes that provides the greatest coverage of training samples. Alternatively, a larger gene set may be chosen either at the user’s request or to enhance classification performance on test (unseen) data. In this scenario, the researcher specifies a value *k*, and the top *k* ranked genes are integrated into the smallest gene subset. The minimum gene subset is included in the final gene set regardless of the genes’ overlap scores. This is because these genes allow the classifier to cover the largest possible portion of the training examples.

**Figure 6:**
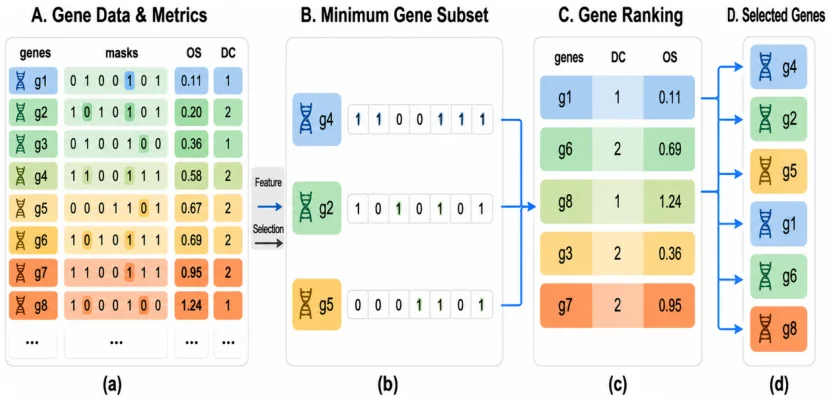
An illustrative example: (**a**) The genes along with their corresponding masks, overlap scores, and dominant class. (**b**) Minimum subset obtained via greedy algorithm. (**c**) Genes are ordered according to their DC and *OS*. (**d**) The final selected gene.

To explain the gene selection process, we use the descriptive example shown in Figure 6a. Every gene is described by its *OS*, a gene mask expressed as a binary string of 0s and 1s, and its DC. Under the binary classification mechanism, each gene is related to either Class 1 or Class 2. For illustration, gene $g_{1}$ has the mask 0100101, indicating that it uniquely identifies the 2nd, 5th, and 7th samples. Its *OS* is 0.11, and its DC is Class 1. To simplify presentation, we arrange the genes in order of increasing *OS*. The primary gene picked through the greedy search technique in the smallest subset is $g_{4}$, due to its feature of possessing the maximum number of bits set to 1 (identical to $g_{6}$ and $g_{7}$) and the minimal overlap score. Next, the genes that provide the best complementary information are $g_{2}$, $g_{5}$, and $g_{6}$, all of which contain the same number of active bits. Among these candidates, $g_{2}$ is picked because of its lowest *OS*. Finally, $g_{5}$ is chosen as it is the only remaining gene with a complementary mask. As a result, the minimum discriminative subset in this example consists of three genes, as illustrated in Figure 6b.

The remaining genes are classified by their DC and arranged by *OS* in ascending order. The gene rank is generated by picking the top-most gene from every DC in a round-robin sequence (e.g., $g_{1}$ from Class 1, $g_{6}$ from Class 2, $g_{3}$ from Class 1, and so on), as shown in Figure 6c. When six genes are needed for biological analysis, the three highest-ranked genes are integrated to the three genes in the smallest subset. Figure 6d shows the final selected gene set.

Hence, the RMP framework can be summarized by the following general selection principle. For each predictor, the method first determines class-specific expression intervals and constructs a binary mask representing unique sample coverage. Robust core intervals are based on the median and $Q_{n}$ estimator to quantify class overlap. Finally, genes are selected using maximum incremental sample coverage, with overlap score used as the deterministic tie-breaking criterion and dominant class used to maintain class-wise representation. Hence, the framework is governed by a fixed sequence of mathematical rules rather than dataset-specific empirical decisions. This structure allows the same procedure to be reproduced on independent datasets and extended to other high-dimensional binary classification problems.

To ensure a fair assessment of the proposed RMP framework, its performance is evaluated using the same datasets, training–testing partitions, classifiers, and evaluation criterion as those used for the competing feature selection methods. The comparison considers not only the Classification Error Rate (CER), but also the stability of the selected gene subsets across different numbers of selected genes. Accordingly, we assess RMP effectiveness from two complementary perspectives: predictive performance and selection stability. We therefore interpret the results in terms of comparative performance rather than assuming the proposed method is universally superior.

## 3. Results

We evaluated the Robust Masked Painter technique on four publicly available standard gene expression datasets and compared it with state-of-the-art feature selection techniques. We also assessed the proposed method on simulated datasets to examine its robustness across different scenarios. We measured performance by classification accuracy. Table 1 provides a short overview of the datasets used in this study.

**Figure 7:**
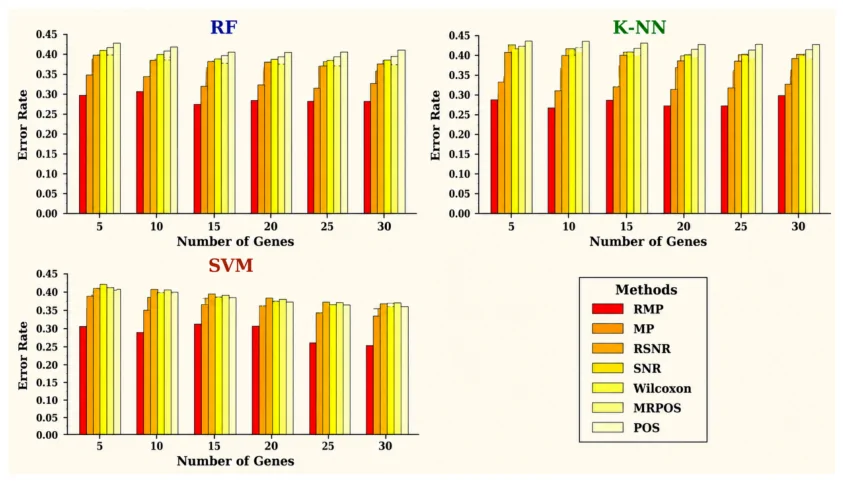
CER of the methods for different numbers of genes for the Tumors-C dataset.

This section evaluates the proposed method against MP, RMPOS, POS, Wilcoxon, RSNR, and SNR on four public gene expression datasets. Utilizing $\frac{70}{30}$ train–test splits and classifiers (K-NN, RF, SVM), the proposed technique achieved lower error rates and higher sensitivity across varying gene counts (5–30). Findings presented in bold in the tables and bar charts demonstrate consistent outperformance compared to the other techniques. Table 2 summarizes CER for the Tumors-C dataset using RF, K-NN, and SVM classifiers. The proposed method demonstrates competitive and, in several datasets and experimental settings, improved classification performance compared with the competing feature selection methods. For RF and SVM, it achieves the lowest error rates in most cases, confirming its superior accuracy. With K-NN, while MP performs slightly better at 10 and 15 genes, the proposed method excels at 5, 20, 25, and 30 genes**.** Figure 7 visually supports these findings, highlighting the robustness of the proposed method across classifiers and gene subset sizes.

**Figure 8:**
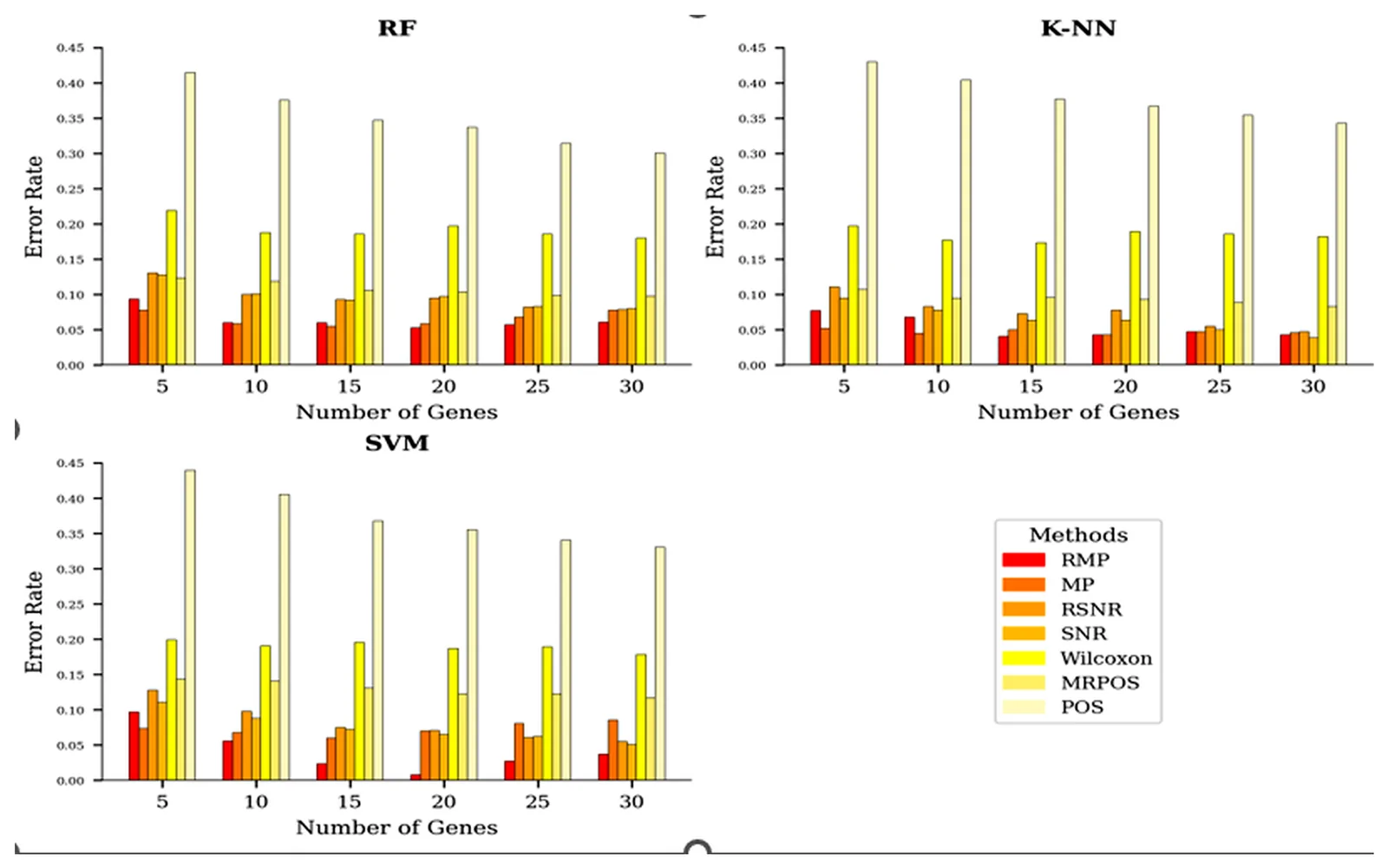
CER of the methods for different numbers of genes for the Lymphoma-Cancer dataset.

Table 3 presents CER for the Lymphoma-Cancer dataset using RF, K-NN, and SVM classifiers. With RF, the MP method yields the lowest errors at smaller gene counts (5 to 15), while the proposed method performs best at 20 to 30 genes. In K-NN, MP leads at five and 10 genes, but the proposed method outperforms others for 15 to 25 genes, whereas SNR performs best at 30 genes. With SVM, the proposed method consistently achieves the lowest error rates across most gene subset sizes, especially with larger sets. Figure 8 visually confirms these results, highlighting the proposed method’s strong and consistent performance.

**Figure 9:**
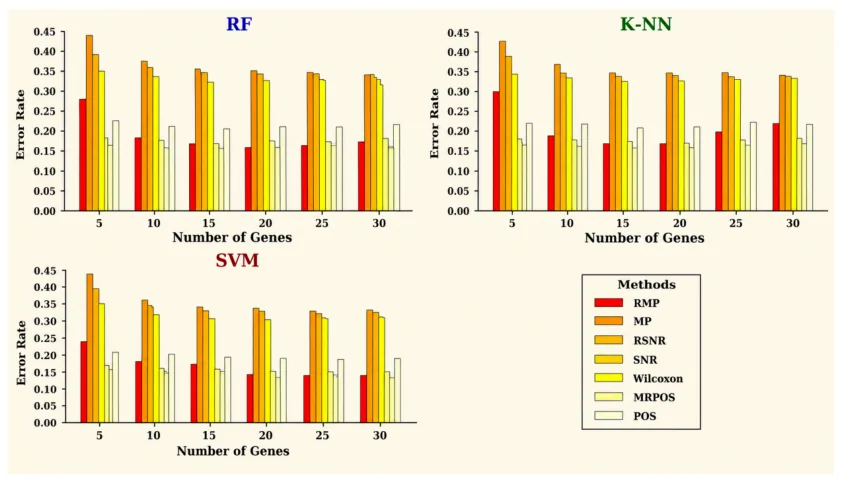
CER of the methods for different numbers of genes for the GSE-4045 dataset.

Table 4 shows Classification Error Rates on the GSE-4045 dataset using RF, K-NN, and SVM classifiers. With RF, MRPOS yields the lowest errors at smaller gene counts (five and 10), while the proposed method performs best at 15 to 30 genes. In K-NN, the MRPOS method yields the lowest errors at 5, 10, 25, and 30 gene counts, while the proposed method performs best at 15 and 20 genes. With SVM, the proposed method consistently achieves the lowest error rates across most gene subset sizes, especially with larger sets. Figure 9 clearly shows these findings, highlighting the proposed method’s consistent superiority, especially with larger gene sets.

**Figure 10:**
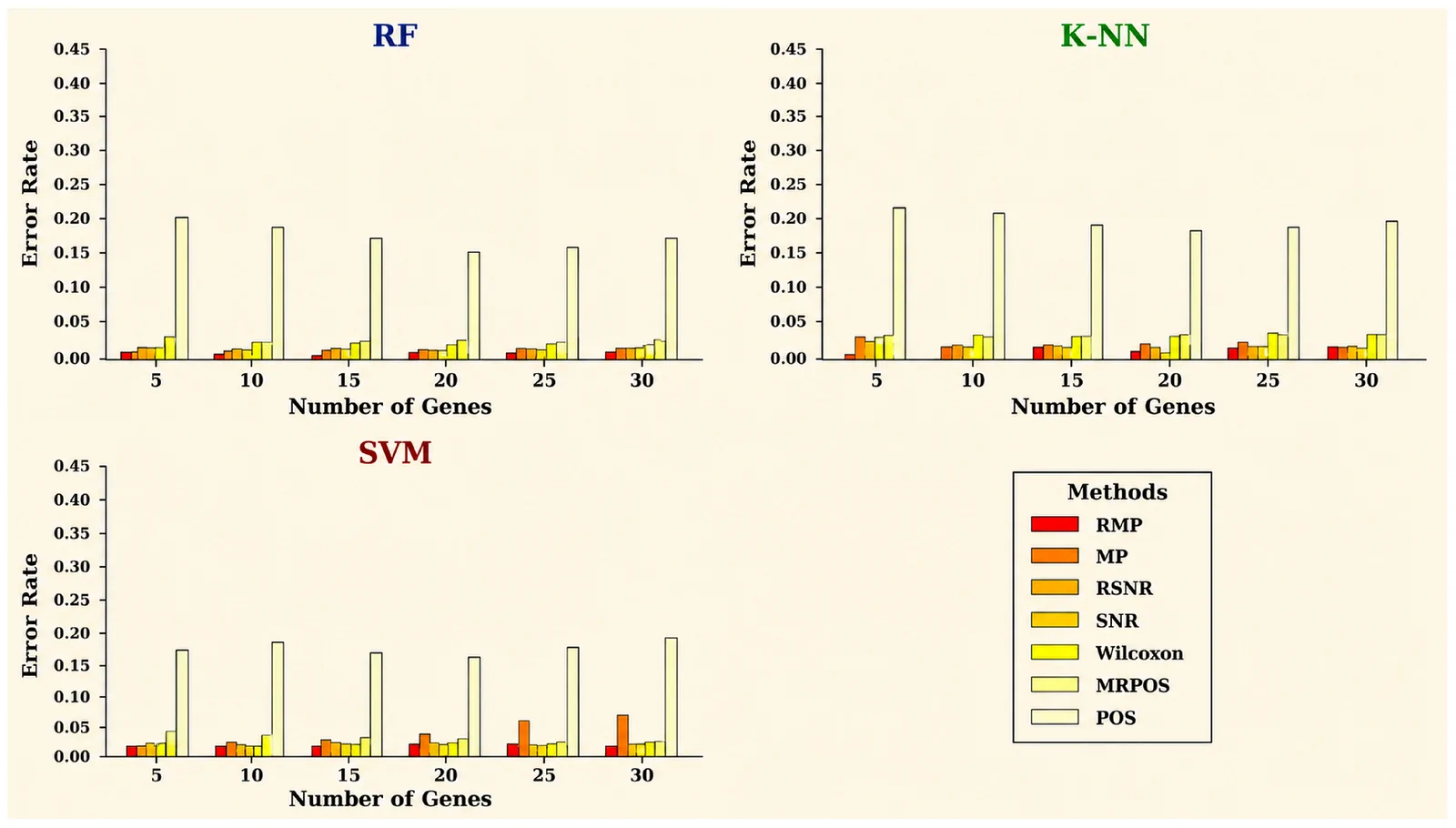
CER of the methods for different numbers of genes for the Lung-Cancer dataset.

Table 5 presents the CER for the Lung-Cancer dataset using RF, K-NN, and SVM classifiers. With RF, MP performs best at five genes, while the proposed technique achieves the lowest error rates from 10 to 30 genes. In K-NN, the proposed technique consistently outperforms others at majority gene counts. For SVM, MP, and Wilcoxon, they perform well at lower gene counts, while the proposed method achieves the best results at 30 genes. Figure 10 indicates the proposed method’s overall effectiveness, particularly as gene count increases.

### 3.1. Statistical Test for Significance

A non-parametric Kruskal–Wallis (KW) test was employed to examine whether statistically significant differences existed among the CER obtained from the different feature selection techniques. The KW test is a rank-based non-parametric method used when the normality assumption is not satisfied. For each dataset, we evaluated the feature selection methods under the same experimental settings, including gene-count levels and classifiers. Therefore, we interpret the statistical results in light of the common experimental structure rather than assuming that all reported CER values are completely independent.

Table 6 summarizes the KW test results for the Classification Error Rates obtained from the different feature selection techniques across the four benchmark datasets. The test results indicate statistically significant differences among the evaluated methods for all four datasets, with p<0.001 in each case. These results provide statistical evidence that the observed differences in CER among the feature selection methods are unlikely to be attributable solely to random variation under the applied experimental settings.

Post hoc comparisons were subsequently examined to identify the methods contributing to the observed differences. For the Tumors-C dataset, RMP showed significantly lower CER than MP, RSNR, SNR, Wilcoxon, MRPOS, and POS. For the Lymphoma-Cancer dataset, RMP showed comparable performance to MP while achieving lower CER than RSNR, SNR, Wilcoxon, MRPOS, and POS. For the GSE-4045 dataset, RMP generally achieved lower CER than the competing feature selection methods. For the Lung-Cancer dataset, RMP achieved lower CER than MP, Wilcoxon, MRPOS, and POS, while its performance was comparable to RSNR and SNR. Hence, the statistical and descriptive results indicate that RMP provides competitive and generally improved classification performance across the evaluated datasets and experimental settings. Nevertheless, because the results were obtained under common classifiers and gene-count settings, some dependence exists among the evaluated observations. Therefore, the statistical analysis is considered complementary evidence for the observed performance differences rather than a standalone demonstration of superiority.

### 3.2. Simulation

This section outlines two different simulation scenarios. The first scenario presents a data-generating process that may limit performance by introducing 10% outliers. The second scenario is structured to showcase the effectiveness by integrating 20% outliers to evaluate the robustness of the proposed technique. For contamination, outliers are incorporated from a Normal (20, 60) distribution into the top twenty genes (*k* = 20), identified as most significant based on their coefficient values from the preceding model. The contaminated features are drawn from a Normal (5, 10) distribution. This procedure yields a simulated dataset containing of N=100 observations and p=120 features. We construct two distinct models using a similar mechanism, with one key distinction: in the first model, we introduce 10% outliers in the significant features, whereas in the second model, we introduce 20% outliers in the significant features.

For each scenario, the predictor matrix consists of independently and identically distributed Normal (0,1) and Uniform (0,1) random variables. A coefficient vector β is generated from a Uniform (−5,5) distribution, and the linear predictor is defined as

(14) $$\eta_{i}=X_{i}\beta .$$

The class probability is then calculated as

(15) $$p_{i}=\frac{\exp\left(b\eta_{i}-a\right)}{1+\exp\left(b\eta_{i}-a\right)}.$$

where the parameters *a* and *b* are both set to 1.5. The binary class label is subsequently assigned according to

(16) $$Y_{i}=\left\{\begin{array}{cc} 1, & \mathrm{if}\;p_{i}\geq 0.5,\\ 0, & \mathrm{otherwise}. \end{array}\right.$$

We perform 500 independent simulation iterations. In each iteration, we apply the proposed and competing feature selection methods under the same experimental settings used for the benchmark gene expression datasets.

In each scenario, the binary response variable Y∼Bernoulli(p) is generated based on an N×p matrix *X*. The elements of X are independent and identically distributed (i.i.d.) observations drawn from Uniform (0, 1) and Normal (0, 1) distributions, via the following expression:

(17) $$p\left(\frac{Y}{X}\right)=\frac{exp(b*X-a)}{1+exp(b*X-a)}$$

We set the parameters *a* and *b* to 1.5. A coefficient vector β is generated from a Uniform (−5, 5) distribution to fit the linear predictor, given by:

(18) Y=Xβ+ϵ

This paper executes a total of five hundred iterations for data simulation. The algorithms run under the same experimental conditions specified for the benchmark or standard datasets. The findings for all techniques presented in this article are derived exclusively on three classifiers: RF, K-NN, and SVM. Table 7 and Table 8 present the results for two scenarios: data with 10% outliers and data with 20% outliers, respectively. Table 7 indicates that the proposed method is less effective compared to the rival methods when the dataset has a smaller percentage of outliers. Nevertheless, as the percentage of outliers increases, the proposed method tends to beat the others, as illustrated in Table 8.

**Figure 11:**
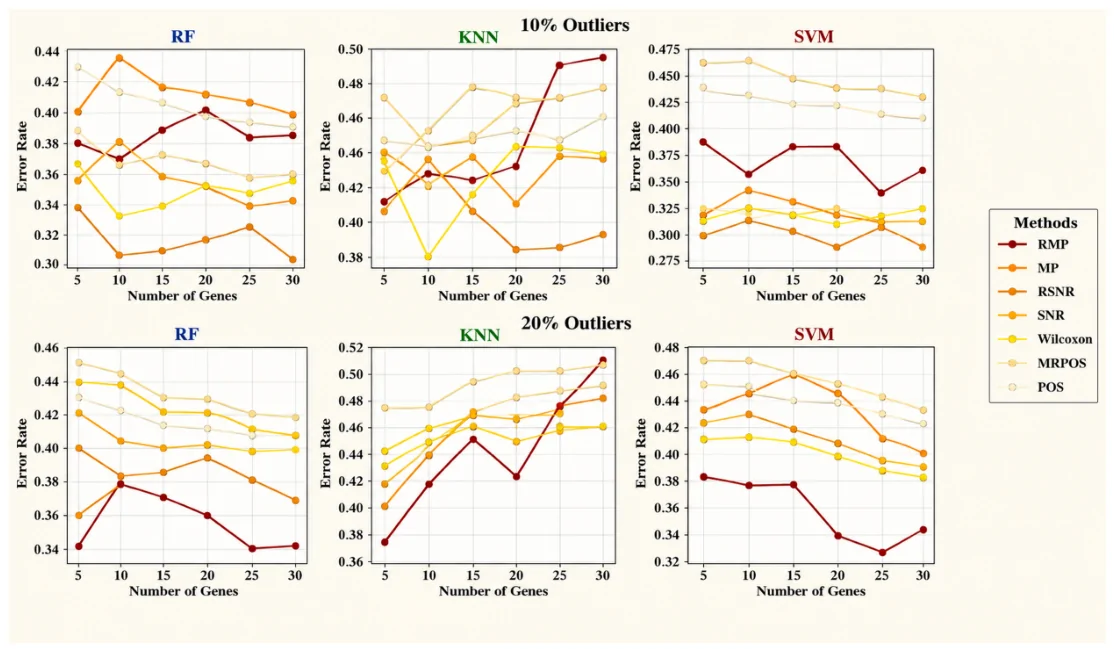
The CER of the all considered classifiers for different subsets of genes for the datasets: (**top panel**) when there is 10% contamination in the features, (**bottom panel**) when there is 20% contamination in the features.

Figure 11 displays the CER of the various techniques for different subsets of genes for the datasets: (I) when there is 10% contamination in the features, (II) when there is 20% contamination in the features.

The comparative results indicate that RMP achieves lower CER than the competing feature selection methods in a substantial proportion of the evaluated experimental settings. However, the results do not imply universal dominance across all datasets and classifiers. Rather, they demonstrate that the proposed robust selection mechanism provides a competitive and frequently advantageous alternative, particularly in settings affected by noise, outliers, and overlapping class distributions. The stability analysis further supports the reliability of the selected gene subsets.

## 4. Discussion

Outliers critically influence the reliability and stability of feature selection techniques, particularly in high-dimensional biological data such as gene expression profiles. These influential data points can stem from technical glitches, experimental variability, or essential biological diversity, and they can significantly disrupt a technique’s ability to identify appropriate features. As the percentage of outliers grows, the efficacy of several traditional feature selection approaches often declines. In the current study, the existing technique, while successful under clear conditions and with a lower outlier percentage (10%), becomes more sensitive to higher outlier percentages, yielding poor results. For illustration, MP achieves lower error rates across various numbers of genes (5–30). However, the proposed method outperforms MP and other existing approaches only when the dataset contains a higher proportion of outliers (20%), showing greater efficiency under challenging masked environments. Overall, the proposed methodology outperforms in most cases by applying robust statistical principles. These results highlight the importance of robustness in feature selection for noisy or contaminated datasets and suggest that future enhancements should focus on outlier-resistant approaches to ensure stable, reliable performance across varying levels of data contamination.

Future studies might integrate additional robust statistical measures, such as MAD and the $S_{n}$ statistic, to improve resistance to noise and outliers. The proposed method could also be extended beyond binary classification to manage multi-class classification problems, which are frequently encountered in complex biological studies. Moreover, the methodology could be adapted for continuous response variables, broadening its applicability to regression-based genomic analyses. Despite the proposed procedure’s effectiveness in identifying highly discriminative genes, two or more selected genes may still convey overlapping information. This redundancy could potentially reduce the interpretability and efficiency of the final gene set. A practical solution is to combine the proposed selection strategy with the LASSO technique, which can further refine the feature set by enforcing sparsity. Alternatively, the proposed method may be applied within each cluster after grouping genes into distinct clusters, allowing selection of representative, non-redundant genes from each cluster.

## 5. Conclusions

In this study, we introduced an RMP method for identifying the most discriminative genes in microarray datasets, while using Median and Rousseeuw & Croux statistic ${(Q}_{n})$ as robust measures of location and variability. Using three classifiers, the study examined outliers in gene expression values that may negatively affect classification performance in high-dimensional gene expression datasets. Feature selection techniques may produce misleading rankings when outliers exist in expression levels. Using a minimum set of genes selected via a greedy search strategy, we construct robust core intervals for classes in binary classification problems to reduce the influence of outliers in gene expression data. Since genes with minimal or no overlap between class intervals are considered highly discriminative, the *OS* is used to measure class separation. For each gene, we also compute DC and arrange genes in increasing order of *OS* within the DC framework. Based on DC and *OS*, two mutually exclusive gene sets are formed. We then apply a round-robin ranking approach using DC and *OS* to generate a ranked list of genes. The greedy search strategy excludes the selected genes from this ranking process. The final gene set combines the greedily selected genes with the highest-ranked genes from the ordered list, while removing the remaining genes to reduce dimensionality. We evaluate the proposed strategy against other popular feature selection methods using RF, K-NN, and SVM classifiers. The results show that the proposed method outperforms competing approaches in most cases in terms of CER. In addition, we assess stability across different numbers of selected genes, and the findings indicate that the proposed method remains more stable than other methods.

Although RMP demonstrates competitive and frequently improved performance over the considered feature selection methods, its superiority should not be interpreted as universal across all datasets, classifiers, or experimental conditions. Further evaluation on additional high-dimensional genomic datasets would help assess its broader generalizability. The present study primarily evaluates classification performance using the CER. Although this measure provides an overall assessment of classification errors, it may not fully reflect class-specific performance in imbalanced datasets. Therefore, sensitivity, specificity, balanced accuracy, MCC, ROC-AUC, and PR-AUC would provide additional perspectives for evaluating the proposed method. A more comprehensive evaluation of these measures, together with uncertainty estimates, is an important direction for future research.

## Figures and Tables

**Table 1:** A short overview of all datasets.

Dataset	Samples	Genes	Class Sizes
Tumors-C	60	7129	21/39
Lymphoma-Cancer	45	4026	23/22
GSE-4045	37	22,215	29/8
Lung-Cancer	148	12,534	134/14

**Table 2:** Classification Error Rate (CER) yielded by various feature selection techniques for the Tumors-C dataset using RF, K-NN, and SVM classifiers.

Genes	RMP	MP	RSNR	SNR	Wilcoxon	MRPOS	POS
Random Forest (RF)							
5	0.314	0.352	0.414	0.417	0.413	0.392	0.426
10	0.331	0.373	0.403	0.405	0.390	0.378	0.422
15	0.279	0.341	0.399	0.392	0.390	0.366	0.407
20	0.286	0.359	0.391	0.391	0.382	0.364	0.404
25	0.284	0.327	0.388	0.383	0.383	0.365	0.401
30	0.295	0.335	0.381	0.382	0.381	0.364	0.391
K-Nearest Neighbors (K-NN)							
5	0.292	0.302	0.419	0.435	0.412	0.392	0.428
10	0.306	0.236	0.405	0.420	0.392	0.374	0.429
15	0.310	0.254	0.401	0.409	0.380	0.371	0.426
20	0.289	0.295	0.395	0.396	0.375	0.367	0.427
25	0.287	0.308	0.396	0.405	0.379	0.367	0.420
30	0.315	0.323	0.391	0.402	0.369	0.374	0.421
Support Vector Machine (SVM)							
5	0.303	0.409	0.398	0.418	0.401	0.381	0.385
10	0.301	0.371	0.402	0.402	0.385	0.381	0.386
15	0.328	0.348	0.384	0.384	0.378	0.365	0.375
20	0.346	0.379	0.376	0.376	0.363	0.362	0.373
25	0.262	0.394	0.366	0.366	0.370	0.355	0.375
30	0.244	0.379	0.360	0.360	0.363	0.357	0.367

**Table 3:** Classification Error Rate (CER) yielded by various feature selection techniques for the Lymphoma Cancer dataset using RF, K-NN, and SVM classifiers.

Genes	RMP	MP	RSNR	SNR	Wilcoxon	MRPOS	POS
Random Forest (RF)							
5	0.094	0.078	0.131	0.128	0.219	0.124	0.415
10	0.060	0.059	0.100	0.101	0.188	0.119	0.376
15	0.060	0.055	0.093	0.092	0.186	0.106	0.347
20	0.053	0.059	0.095	0.097	0.197	0.104	0.337
25	0.057	0.068	0.082	0.083	0.186	0.099	0.314
30	0.061	0.078	0.079	0.080	0.180	0.098	0.301
K-Nearest Neighbors (K-NN)							
5	0.077	0.052	0.111	0.095	0.197	0.107	0.430
10	0.068	0.045	0.083	0.078	0.177	0.095	0.404
15	0.041	0.050	0.073	0.064	0.174	0.096	0.377
20	0.043	0.043	0.078	0.064	0.189	0.093	0.367
25	0.047	0.047	0.055	0.050	0.186	0.089	0.354
30	0.043	0.046	0.047	0.039	0.182	0.083	0.343
Support Vector Machine (SVM)							
5	0.097	0.074	0.128	0.111	0.199	0.144	0.440
10	0.056	0.068	0.098	0.088	0.191	0.141	0.406
15	0.024	0.060	0.075	0.072	0.196	0.131	0.368
20	0.008	0.070	0.071	0.065	0.187	0.123	0.356
25	0.027	0.081	0.061	0.062	0.189	0.123	0.341
30	0.037	0.086	0.055	0.051	0.179	0.117	0.331

**Table 4:** Classification Error Rate (CER) yielded by various feature selection techniques for the GSE 4045 dataset using RF, K-NN, and SVM classifiers.

Genes	RMP	MP	RSNR	SNR	Wilcoxon	MRPOS	POS
Random Forest (RF)							
5	0.262	0.257	0.443	0.384	0.357	0.171	0.245
10	0.177	0.233	0.386	0.360	0.355	0.170	0.234
15	0.151	0.227	0.359	0.352	0.342	0.155	0.207
20	0.141	0.227	0.363	0.356	0.355	0.179	0.203
25	0.141	0.225	0.354	0.348	0.343	0.177	0.210
30	0.143	0.225	0.342	0.333	0.339	0.169	0.224
K-Nearest Neighbors (K-NN)							
5	0.304	0.274	0.408	0.371	0.351	0.183	0.241
10	0.188	0.244	0.359	0.337	0.344	0.179	0.239
15	0.159	0.241	0.337	0.326	0.352	0.168	0.221
20	0.138	0.247	0.348	0.334	0.357	0.178	0.218
25	0.203	0.236	0.337	0.329	0.349	0.175	0.216
30	0.209	0.235	0.324	0.324	0.345	0.176	0.229
Support Vector Machine (SVM)							
5	0.255	0.226	0.447	0.374	0.343	0.169	0.221
10	0.187	0.246	0.398	0.364	0.350	0.167	0.226
15	0.184	0.243	0.371	0.357	0.348	0.152	0.210
20	0.141	0.248	0.382	0.364	0.366	0.165	0.210
25	0.150	0.256	0.362	0.368	0.361	0.162	0.215
30	0.133	0.242	0.361	0.351	0.354	0.156	0.231

**Table 5:** Classification Error Rate (CER) yielded by various feature selection techniques for the Lung-Cancer dataset using RF, K-NN, and SVM classifiers.

Genes	RMP	MP	RSNR	SNR	Wilcoxon	MRPOS	POS
Random Forest (RF)							
5	0.010	0.009	0.019	0.019	0.032	0.024	0.245
10	0.005	0.011	0.016	0.016	0.025	0.026	0.234
15	0.006	0.014	0.016	0.016	0.024	0.026	0.207
20	0.006	0.014	0.014	0.014	0.021	0.025	0.203
25	0.007	0.016	0.016	0.015	0.022	0.024	0.210
30	0.009	0.016	0.015	0.016	0.020	0.024	0.224
K-Nearest Neighbors (K-NN)							
5	0.002	0.008	0.025	0.023	0.036	0.031	0.241
10	0.001	0.018	0.022	0.021	0.037	0.033	0.239
15	0.016	0.020	0.022	0.020	0.035	0.035	0.221
20	0.009	0.024	0.020	0.010	0.036	0.037	0.218
25	0.019	0.023	0.020	0.020	0.040	0.038	0.216
30	0.020	0.018	0.020	0.019	0.038	0.037	0.229
Support Vector Machine (SVM)							
5	0.023	0.022	0.028	0.023	0.032	0.056	0.221
10	0.023	0.028	0.025	0.022	0.021	0.046	0.226
15	0.024	0.034	0.027	0.026	0.024	0.035	0.210
20	0.023	0.032	0.024	0.023	0.024	0.030	0.210
25	0.023	0.061	0.021	0.020	0.023	0.025	0.215
30	0.018	0.074	0.022	0.022	0.024	0.024	0.231

**Table 6:** Kruskal–Wallis test results comparing CER across the feature selection techniques.

Dataset	Kruskal–Wallis $\chi^{2}$	*p*-Value
Tumors-C	64.620	<0.001
Lymphoma-Cancer	82.976	<0.001
GSE-4045	87.106	<0.001
Lung-Cancer	62.604	<0.001

**Table 7:** Classification Error Rate (CER) yielded by various feature selection techniques for the uploaded dataset with 10% outliers using RF, K-NN, and SVM classifiers.

Genes	RMP	MP	RSNR	SNR	Wilcoxon	MRPOS	POS
Random Forest (RF)							
5	0.380	0.333	0.353	0.401	0.365	0.389	0.442
10	0.369	0.300	0.382	0.444	0.329	0.365	0.417
15	0.390	0.302	0.356	0.418	0.335	0.372	0.409
20	0.406	0.310	0.348	0.416	0.350	0.366	0.399
25	0.384	0.320	0.336	0.413	0.345	0.355	0.394
30	0.387	0.293	0.339	0.403	0.354	0.361	0.392
K-Nearest Neighbors (K-NN)							
5	0.412	0.407	0.445	0.478	0.440	0.431	0.448
10	0.430	0.438	0.423	0.445	0.376	0.453	0.445
15	0.426	0.407	0.440	0.448	0.418	0.482	0.452
20	0.434	0.383	0.410	0.469	0.445	0.475	0.454
25	0.492	0.385	0.440	0.474	0.444	0.474	0.449
30	0.496	0.393	0.438	0.480	0.440	0.480	0.463
Support Vector Machine (SVM)							
5	0.390	0.316	0.318	0.418	0.320	0.370	0.471
10	0.356	0.326	0.395	0.461	0.320	0.362	0.444
15	0.381	0.315	0.366	0.437	0.321	0.355	0.439
20	0.382	0.293	0.327	0.431	0.317	0.353	0.421
25	0.340	0.321	0.303	0.429	0.310	0.341	0.411
30	0.360	0.273	0.302	0.424	0.319	0.356	0.404

**Table 8:** CER by SVM, K-NN, and RF classifiers on simulated data with 20% outliers in the key features.

Genes	RMP	MP	RSNR	SNR	Wilcoxon	MRPOS	POS
Random Forest (RF)							
5	0.331	0.363	0.380	0.435	0.452	0.437	0.467
10	0.363	0.365	0.383	0.411	0.444	0.383	0.453
15	0.360	0.381	0.380	0.410	0.426	0.386	0.429
20	0.347	0.397	0.367	0.405	0.427	0.387	0.420
25	0.329	0.374	0.369	0.399	0.420	0.381	0.421
30	0.330	0.348	0.359	0.404	0.414	0.375	0.411
K-Nearest Neighbors (K-NN)							
5	0.388	0.384	0.421	0.437	0.455	0.457	0.481
10	0.426	0.426	0.455	0.452	0.470	0.483	0.482
15	0.465	0.482	0.465	0.485	0.478	0.502	0.480
20	0.436	0.479	0.463	0.486	0.465	0.515	0.493
25	0.495	0.493	0.464	0.493	0.471	0.515	0.494
30	0.497	0.526	0.465	0.501	0.467	0.521	0.485
Support Vector Machine (SVM)							
5	0.393	0.422	0.433	0.435	0.444	0.451	0.479
10	0.385	0.441	0.447	0.408	0.415	0.456	0.479
15	0.379	0.469	0.428	0.399	0.412	0.459	0.449
20	0.327	0.463	0.389	0.391	0.414	0.445	0.445
25	0.320	0.380	0.377	0.386	0.412	0.420	0.445
30	0.346	0.379	0.360	0.380	0.409	0.406	0.427

## Data Availability

The gene expression datasets analyzed in this study are publicly available through the OpenML repository (https://www.openml.org), including the Tumors-C dataset. We obtained the GSE4045 dataset from the National Center for Biotechnology Information (NCBI) Gene Expression Omnibus (GEO) database. All other data supporting the findings of this study are provided within the manuscript.
